# Inhibiting Disulfide Bonding in Truncated Tau297–391 Results in Enhanced Self-Assembly of Tau into Seed-Competent Assemblies

**DOI:** 10.1021/acschemneuro.5c00639

**Published:** 2025-12-10

**Authors:** Sebastian S. Oakley, Karen E. Marshall, Georg Meisl, Alice Copsey, Mahmoud B. Maina, Robert Milton, Thomas Vorley, John M. D. Storey, Charles R. Harrington, Claude M. Wischik, Wei-Feng Xue, Louise C. Serpell

**Affiliations:** † Sussex Neuroscience, School of Life Sciences, 1948 University of Sussex, Falmer, East Sussex BN1 9QG, U.K.; ‡ Biomedical Science Research and Training Centre, Yobe State University, Damaturu, Yobe State 620101, Nigeria; § Yusuf Hamied Department of Chemistry, 2152 University of Cambridge, Lensfield Road, Cambridge, Cambs CB2 1EW, U.K.; ∥ Institute of Medicine, Medical Sciences and Nutrition, 1019 University of Aberdeen, Aberdeen AB24 3FX, U.K.; ⊥ Department of Chemistry, University of Aberdeen, Aberdeen AB24 3FX, U.K.; # TauRx Therapeutics Ltd., 395 King street, Aberdeen AB24 5RP, U.K.; ∇ School of Natural Science, 2240 University of Kent, Canterbury CT2 7NJ, U.K.

**Keywords:** tau, self-assembly, nucleation, dGAE, disulfide bonding, tau seeding

## Abstract

Tau undergoes fibrillogenesis in a group of neurodegenerative diseases, termed tauopathies. Each tauopathy is characterized by tau fibrils with disease-specific conformations, highlighting the complexity of tau self-assembly. This has led to debate surrounding the precise mechanisms that govern the self-assembly of tau in disease, especially the involvement of disulfide bonding (DSB) between cysteine residues. In this study, we use a truncated form of tau, dGAE, capable of forming filaments identical to those in disease. We reveal the impact of DSB on dGAE assembly and propagation by resolving the global mechanisms that dominate its assembly. We found evidence of surface-mediated secondary nucleation and fragmentation being active in dGAE assembly. The inhibition of DSB during dGAE assembly leads to an enhanced aggregation rate through a reduced lag phase but with no effect on the global assembly mechanisms. We suggest this is due to the formation of a dominant, seed-competent species in the absence of DSB that facilitates elongation and secondary nucleation, resulting in enhanced assembly. *In vitro* seeding assays reveal the recruitment of endogenous tau in a cell model only when using dGAE species formed under conditions that inhibit DSB. Our results further support the use of the *in vitro* dGAE tau aggregation model for investigating the mechanism of tau assembly, show the effect of varying conditions on tau assembly, and how these conditions affect the resultant species. Further studies may utilize dGAE and its aggregates to investigate tau seeding, propagation, and to highlight or test potential targets for therapies that reduce the spread of pathological tau throughout the brain.

## Graphical abstract

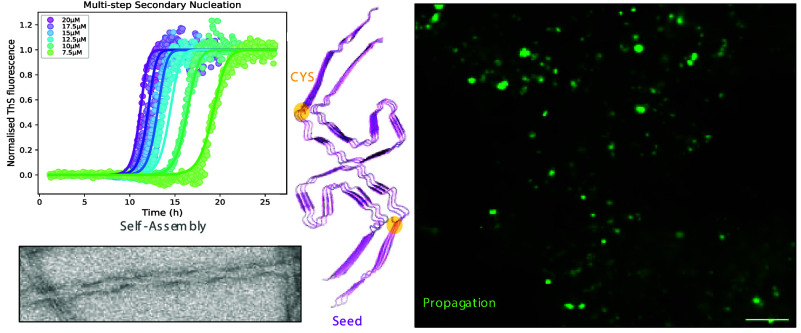

## Introduction

Tauopathy is a collective term for a group of neurodegenerative diseases that are characterized by the deposition of abnormal tau aggregates throughout the brain. These diseases include Alzheimer’s disease (AD), frontal temporal dementia (FTD), chronic traumatic encephalopathy (CTE), and corticobasal degeneration (CBD).^1^ Tau is a microtubule-associated protein involved in promoting and stabilizing the microtubule network.^2^ However, in the brains of patients with tauopathies, tau undergoes a pathological self-assembly process to form highly ordered amyloid fibrils.^3^ These tau amyloid fibrils are strongly associated with neurodegeneration, cognitive decline, and clinical dementia.^4^ Recent cryo-electron microscopy (cryo-EM) studies have resolved the atomic structure of the tau filament structures associated with different tauopathies^5–7^ revealing that, in each disorder, tau self-assembles to form disease-specific conformations. Despite this, all filament structures resolved to date show that a similar region of tau associates to form the cross-β core and that this is comprised predominantly of the imperfect repeat region.^8^ This illustrates the complex polymorphic capacity of tau to form multiple specific atomic structures and interactions that are associated with disease-specific and clinically distinct cognitive impairments. Revealing the initial stages and the mechanisms involved in tau self-assembly and how they affect the final fibril structure is crucial for our understanding of tauopathies and in the development of therapeutics.

Amyloid-specific dyes, such as Thioflavin T or S (ThT/ThS), have been used in kinetic assays to analyze the rate of protein self-assembly into filaments for a range of amyloidogenic proteins, such as β_2_-microglobulin (β_2_m),^9^ tau,^10^ α-synuclein,^11^ and amyloid-β.^12^ The traces obtained from ThT/ThS kinetic assays of these proteins have been pivotal in revealing the mechanisms that dominate the process of amyloidogenic protein self-assembly, such as primary or secondary nucleation processes.^9,13,14^ Primary nucleation refers to a monomer-only nucleation process, whereby the assembly of small oligomeric aggregates, or nuclei, depends on the rates of association of monomers and their dissociation back to monomers. Secondary nucleation denotes a fibril surface-dependent nucleation process, where the assembly of nuclei from monomers is catalyzed by pre-existing fibrils formed from the same protein monomer.^13,15^ Fragmentation is a form of secondary process that amplifies fibril formation through repeated division and elongation cycles. Existing aggregates fragment, exposing additional fibril ends that facilitate further elongation.^9,16^ Previous work using Tau304–380 Cys322Ser variant has highlighted the role of an autocatalytic secondary nucleation mechanism in the self-assembly process of tau in the absence of additives.^10^

ThS/T aggregation kinetic assays can also be utilized to explore the role of specific bonds, bonding regions, and specific residues in amyloid assembly. The impact of disulfide bonding (DSB) between cysteine residues for tau self-assembly is still highly debated. With the use of *in vitro* tau aggregation models, such as full-length tau (T40) and K18/K19 fragments templated using heparin, DSB was proposed as an essential step in tau self-assembly and propagation.^17^ However, the ability of these models to produce reliable tau filaments with the same macromolecular structure as those isolated from tauopathies has been disputed by cryo-EM studies.^18^ A truncated form of tau protein corresponding to residues 297–391, termed dGAE, assembles readily in reducing conditions^19,20^ to form twisted filaments that are structurally identical to AD paired helical filaments (PHFs) and CTE type II filaments.^21–23^ Under reducing conditions, cys322 is prevented from forming disulfide bonds, and the variant C322A is useful in determining the contribution of the cystine residue. dGAE is therefore a valuable model for the investigation of tau self-assembly, filament structure, and propagation. Furthermore, it is currently the only tau fragment able to recapitulate the structural details of the core of in vivo filaments from Alzheimer’s patients.

In this study, we utilize ThS kinetics assays to investigate the assembly mechanisms of dGAE and dGAE-C322A, the involvement of DSB in dGAE assembly, and the seeding characteristics of dGAE aggregates in a cellular environment. We show that secondary processes dominate the assembly mechanism of dGAE, with evidence showing that complex surface-mediated secondary nucleation and fragmentation are active in dGAE assembly. In addition, the inhibition of DSB in dGAE aggregation has no effect on the overall global assembly mechanism but significantly enhances the rate of aggregation resulting in a shorter lag phase. Self-assembled species without DSB are significantly better than those with DSB, at accelerating the aggregation process *in vitro* and at recruiting endogenous tau in tau Biosensor cells. This suggests that the presence of DSB in dGAE filaments is detrimental for those aggregates to facilitate the propagation of tau fibril formation and that the inhibition of DSB is an important step in the formation of pathological tau aggregates.

## Materials and Methods

### Purification of Truncated Tau297–391 (dGAE)

dGAE and dGAE-C322A proteins were expressed in *Escherichia coli* BL21 cells grown in 2x Yeast Tryptone (2xYT) media supplemented with ampicillin. Overnight cultures were diluted to an OD600 of 0.01 and grown at 37 °C and 250 rpm until they reached an OD600 of 0.6, at which point protein expression was induced by the addition of isopropyl β-D-1-thiogalactopyranoside (IPTG) to a concentration of 0.4 mM. After 2 h induction, cells were harvested by centrifugation at 7000 rpm/7122*g* for 10 min at 4 °C (Beckman Avanti J-30I, JLA-16.25 rotor). Cell pellets were transferred to 50 mL Falcon tubes in 0.9% NaCl, and cells were collected by centrifugation at 4500 rpm/2490*g* for 10 min at 4 °C (Beckman Avanti J-30I, JA 25.50 rotor). Cells from 4 L of expression culture were resuspended in 80 mL of lysis buffer (50 mM MES, pH 6.25) with 1 mM ethylenediaminetetraacetic acid (EDTA), 1 mM dithiothreitol (DTT) supplemented with EDTA-free protease inhibitors (Roche, 04693159001). Cells were lysed using 3 min sonication (5s on/5s off) at 50% amplitude with a 13 mm probe on ice and centrifuged at 10,000 rpm/10,600*g* (Thermo Scientific Heraeus Multifuge 3S-R) to remove intact cells and cell debris. NaCl was added to the supernatant at 1.75 g per 40 mL plus DTT to a final concentration of 1 mM. The cell suspension was boiled for 5 min to precipitate proteins, which were removed by centrifugation at 10,000 rpm/10,600*g* for 10 min at 4 °C. The dGAE-containing supernatant was dialyzed overnight into MES buffer (50 mM MES (pH 6.25), 1 mM ethylene glycol-bis­(2-aminoethyl ether)-*N*,*N*,*N*′,*N*′-tetraacetic acid (EGTA), 5 mM EDTA, 0.2 mM MgCl_2_, and 5 mM mercaptoethanol). dGAE was further purified by passage through a 5 mL HiTrap SP Sepharose column attached to a KTA Fast Protein Liquid Chromatography (FPLC). The column was equilibrated at a flow rate of 1 mL min^–1^ in MES buffer. Protein was bound to the column at a flow rate of 0.5 mL min^–1^ and washed at a flow rate of 1 mL min^–1^ until the absorbance at 280 nm had returned to the baseline. Protein was eluted with a 10 times the column volume of MES buffer supplemented with 1 M KCl, and peak fractions were collected in a 96-deep-well plate. Fractions containing dGAE were pooled, dialyzed overnight into phosphate buffer, and stored at −80 °C. The protein was then diluted in phosphate buffer (PB) (10 mM; pH 7.4) for further experimentation. PB was made by adding 200 mM NaH_2_PO_4_ to 200 mM Na_2_HPO_4_ until pH 7.4 was achieved. This stock concentration (200 mM) was diluted to 10 mM for future experiments.

### ThS Kinetics and Analysis

dGAE monomer was added at varying concentrations (1–20 μM) in degassed phosphate buffer, pH 7.4 with 20 μM Thioflavin S (ThS) in low-bind tubes. ThS is an analogue of ThT and was used instead of ThT because of its higher sensitivity with tau amyloid aggregates. Each sample was transferred to a nonbinding μClear bottom, black 96-well plate (Greiner Bio-One, 655906) with a foil seal to stop evaporation. The plate was incubated in a Molecular Devices SpectraMax i3x plate reader equilibrated at 37 °C with high orbital shaking (469 rpm) and readings taken from the bottom of the plate every 5 min using an excitation filter at 440 nm and an emission filter at 483 nm, to monitor ThS fluorescence intensity. The data were normalized using the initial plateau and final plateau using MATLAB as previously described.^9^ Normalized data were then subjected to global chemical kinetics analysis using Amylofit software^14^ to observe how the data fit with each global model of aggregation.

### Preparation of dGAE Fibrils

dGAE or dGAE-C322A (400 μM) was diluted in 10 mM PB ± 10 mM DTT^21,23^ and incubated at 37 °C while agitating at a speed of 400 rpm in an Eppendorf ThermoMixer for 4d. The samples were then centrifuged at 16,000*g* at 4 °C for 30 min. The supernatant was removed to determine the protein concentration of the supernatant to estimate the protein content in the pellet. Protein concentration was estimated using the Pierce Bicinchoninic Acid (BCA) assay (Thermo Scientific, 23225) and with a reducing agent-compatible BCA kit (Thermo Scientific, 23250) used for the dGAE+DTT sample. PB (10 mM) was added to the pellet to suspend fibrils at a final concentration of 400 μM dGAE. If the samples had DTT in the assembly mixture, these fibrils were washed once with PB, with an additional centrifugation step before final suspension. Sonication was done in a Fisher Scientific water bath sonicator (FB 15051) for 10 min with ice to reduce the effect of heating. To investigate species present throughout assembly, incubation was extended to 7 days and a small sample was removed from the assembly mixture (without centrifuging to obtain aggregated and soluble protein) to be mixed with Laemmli sample buffer (Bio-Rad Laboratories, 1610747) without BME and loaded onto an Any kDa Mini-PROTEAN precast gel (Bio-Rad Laboratories, 4569036) and run at 200 V for 30 min in TRIS-glycine-SDS running buffer. Coomassie stain was applied for 1 h, destained once for 1 h, and again overnight. Gels were imaged on a Li-Cor Odyssey FC imaging system (exposure 30s).

### Transmission Electron Microscopy (TEM)

Electron microscopy grids were prepared by withdrawing 4 μL of sample from assays and placing onto Formvar/carbon-coated 400-mesh copper grids (Agar Scientific, AGS162–4), blotting excess, and then washing with 4 μL of 0.22 μM filtered Milli-Q water. Uranyl acetate (4 μL of 2% in water) was placed on the grid once for 1 min and then blotted, and the grid was allowed to air-dry. TEM projection images were collected using a JEOL JEM1400-Plus Transmission Electron Microscope operated at 120 kV equipped with a Gatan OneView camera (4 × 4k). Images were recorded at 25 fps with drift correction using GMS3.

### SDS-PAGE

Samples were mixed with Laemmli sample buffer (Bio-Rad Laboratories, 1610747) without BME and loaded onto an Any kDa Mini-PROTEAN precast gel (Bio-Rad Laboratories, 4569036) and run at 200 V for 30 min in TRIS-glycine-SDS running buffer. SimplyBlue SafeStain (Invitrogen, 465044) was applied for 1 h, destained with water once for 1 h and again overnight. Gels were imaged on a Li-Cor Odyssey FC imaging system (exposure 30s).

### Circular Dichroism (CD)

dGAE (±DTT), and dGAE-C322A fibrils were spun to separate the supernatant and pellet. The pellet was resuspended in PB to a final concentration of 400 μM. dGAE-DTT fibrils were washed once to remove excess DTT. CD was performed using a Jasco Spectrometer J715, and spectra were collected in triplicate at a maintained temperature of 21 °C. Protein samples of the pellet (60 μL) were placed into 0.2 mm path length quartz cuvettes (Hellma), and data were collected between wavelengths of 180 to 350 nm.

### Proteinase K (PK) Digestion

Fibrils from each condition were diluted to 200 μM with 10 mM PB containing 25 μg/mL of proteinase K (PK: Merck, P2308) (prepared in 50 mM Tris/1 mM CaCl_2_, pH 7.5) and incubated for 1h at 37 °C. The same amount of PK buffer was added to controls without PK. Samples were then subjected to SDS-PAGE and Coomassie staining before being imaged.

### Cell Culture and Fluorescence Aggregation Assay

Tau RD P301S FRET Biosensor cells (here referred to as tau Biosensor cells) from ATCC (CRL-3275)^24^ were grown in Dulbecco’s modified Eagle medium/Nutrient Mixture F-12 (DMEM/F-12, Thermo Scientific, 12634010) supplemented with 10% fetal calf serum, 1% penicillin/streptomycin (P/S), and 1% l-glutamine. Cells were plated at a density of 10,000 cells per well in 150 μL in a 96-well plate and incubated for 2 days. Media was replaced with 10 μM of soluble, fibrils, or sonicated fibrils from each condition (dGAE, dGAE+DTT, and dGAE-C322A) and incubated for 3d at 37 °C in 5% CO_2_. Fibrils and sonicated fibrils were resuspended to 400 μM after centrifugation, meaning 3.75 μL of the isolated sample or PB was added to the media to make the replacing media. dGAE+DTT fibrils were washed with PB to remove DTT from the sample and resuspended in sterile PB without DTT to eliminate the toxicity on the cells. No toxicity was observed in the dGAE+DTT samples when compared with the dGAE or dGAE-C322A samples. Sonicated fibrils were prepared by sonicating for 10 min in an iced sonication bath. 10 min was the optimal time frame to produce a consistent sample. Post 72h of incubation, the plate was imaged using the automated Molecular Devices ImageXpress Pico using FITC filter with 20× objective. Analysis was carried out using the CellReporterXpress software, whereby optimization of the cell counting analysis protocol was adapted to specifically select the punctate fluorescence signal produced as a result of aggregated endogenous tau. Parameters used were as follows: minimum size = 2, maximum size = 10, and intensity = 150. This was optimized to be effective at isolating the fluorescence signal for accurate analysis of aggregated endogenous tau (Supporting Information Figure S7). Imaging and analysis were carried out on the punctate YFP fluorescence; no FRET quantification was performed in this study.

### Data Analysis and Representation

Data and statistical analyses were performed using Microsoft Excel, GraphPad Prism 7, and MATLAB R2022a. All data are expressed as the mean  ±  SEM. When comparing two groups, a form of *t* test was used to determine the statistical significance. When comparing more than two groups, a form of a one-way ANOVA test was used to determine if there is a difference between experimental groups and a control group. The normality and distribution of the data were calculated to decide upon the specific *t* test and one-way ANOVA. Specific details for the individual statistical tests and multiple comparison tests performed can be found in the figure legends. Differences were considered to be statistically significant if *p* < 0.05.

## Results

### dGAE Assembly Is Dominated by Secondary Processes and Is Independent of Disulfide Bonding

To investigate the contribution of DSB to dGAE assembly mechanisms, ThS fluorescence assays were used to monitor the kinetics of self-assembly of dGAE under conditions that allow or inhibit DSB. Conditions for reproducible spontaneous assembly of dGAE in nonreducing and reducing conditions (with 10 mM DTT), or dGAE-C322A in nonreducing conditions were optimized using a SpectraMax i3x plate reader. dGAE-C322A variant was used to inhibit disulfide bonding through the substitution of the only cysteine residue in dGAE. This variant has previously been shown to assemble into filaments.^19^ dGAE samples prepared with a dilution series (1–20 μM) were measured using ThS. Agitation was found to be necessary for reproducible assembly. All data showed a sigmoidal-like appearance with a lag phase, a steep growth phase, and a final plateau (Figure 1). A clear concentration dependence was observed showing faster assembly at higher concentrations, with unreliable assembly observed at a dGAE monomer concentration of below ∼7 μM (data not shown). The data were normalized to the initial baseline and final plateau before uploading to Amylofit software.^14^ The time to reach the midpoint between the initial baseline and final plateau values is known as the half-time, which, when plotted against monomer concentration in a log–log plot, was used to generate the scaling exponent for dGAE assembly in the different conditions (Figure 1a). The straight line of the scaling exponent indicates that the dominant mechanism of aggregation does not change within the monomer concentration range tested. The value of the scaling exponent is close to −1/2 for all conditions (dGAE = −0.517, dGAE+DTT = −0.512, and dGAE-C322A = −0.520), which suggests that either or both fragmentation and saturated secondary nucleation (multistep secondary nucleation) dominate dGAE aggregation, regardless of DSB involvement. To further clarify this, normalized kinetic profiles were plotted together with best-fit global models of aggregation mechanisms. A good fit of the model to the data was observed for both (1) multistep secondary nucleation of monomers mediated by the fibril surface (saturated secondary nucleation) (Figures 1b and 2) fragmentation process with primary nucleation and elongation mediated by the exposed ends of the fibrils (Figure 1c). The necessity of including agitation in the experimental approach may affect the relative contributions of fragmentation and secondary nucleation. For comparison, models for nucleation elongation, secondary nucleation, and saturating elongation and secondary nucleation show a poor fit for each condition (Supporting Information Figure S1). The mean residual error (MRE) calculated by Amylofit is used to measure the goodness of fit to the varying aggregation models (a lower value indicates a better fit to the data). MRE values also suggest that multistep nucleation and elongation with fragmentation are the best fit for the data when compared to the poorly fitting models (Supporting Information Figure S1d).

**Figure 1:**
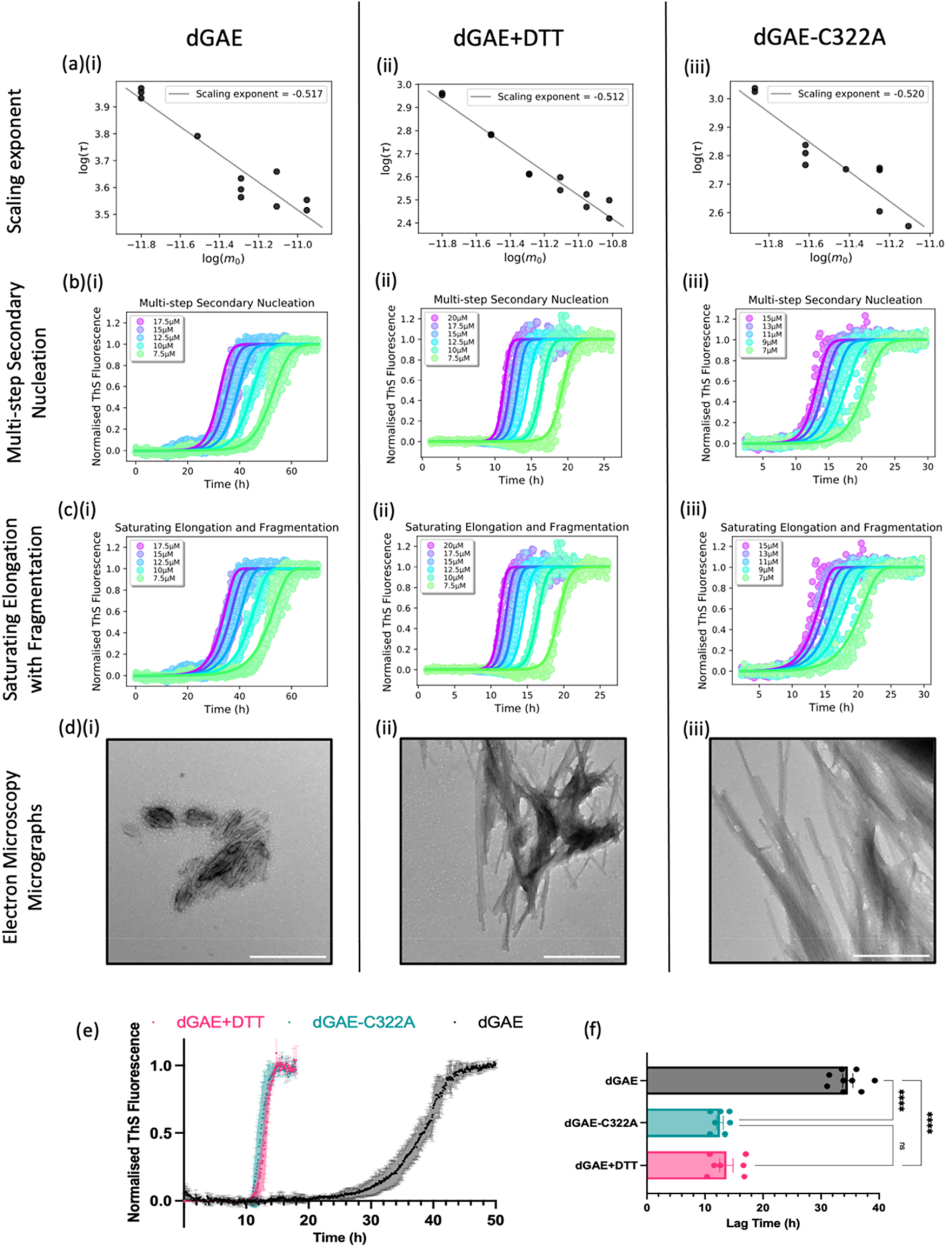
Columns show results arising from (i) dGAE assembled in nonreducing conditions, (ii) dGAE assembled in reducing conditions with 10 mM DTT, and (iii) dGAE-C322A assembled in nonreducing conditions. (a) Scaling exponent of each titration experiment. Normalized kinetic profiles plotted fitted to different models of assembly: (b) multistep secondary nucleation and (c) saturating elongation with fragmentation. (d) Electron micrographs of the resultant assemblies taken when the final plateau has been reached. Scale bars = 500 nm. (e) Comparison of normalized thioflavin-S kinetic profile from one experiment with a starting concentration of 15 μM monomeric dGAE in nonreducing conditions (black), dGAE in reducing conditions with 10 mM DTT (pink), and dGAE-C322A in nonreducing conditions (green). (f) Quantification and comparison of the lag time for each condition; results were taken from 3 independent experiments. [One-way ANOVA showed a significant difference between the groups (*F* = 206.2, *R* ^2^ = 0.9538, *p* < 0.0001). Tukey’s multiple comparisons test showed significance when comparing dGAE (34.60 ± 0.8734 h) with both dGAE+DTT (13.69 ± 1.139 h, *p* < 0.0001) and dGAE-C322A (12.60 ± 0.5575 h, *p* < 0.0001), but there was no significant difference between dGAE+DTT and dGAE-C322A.].

**Figure 2:**
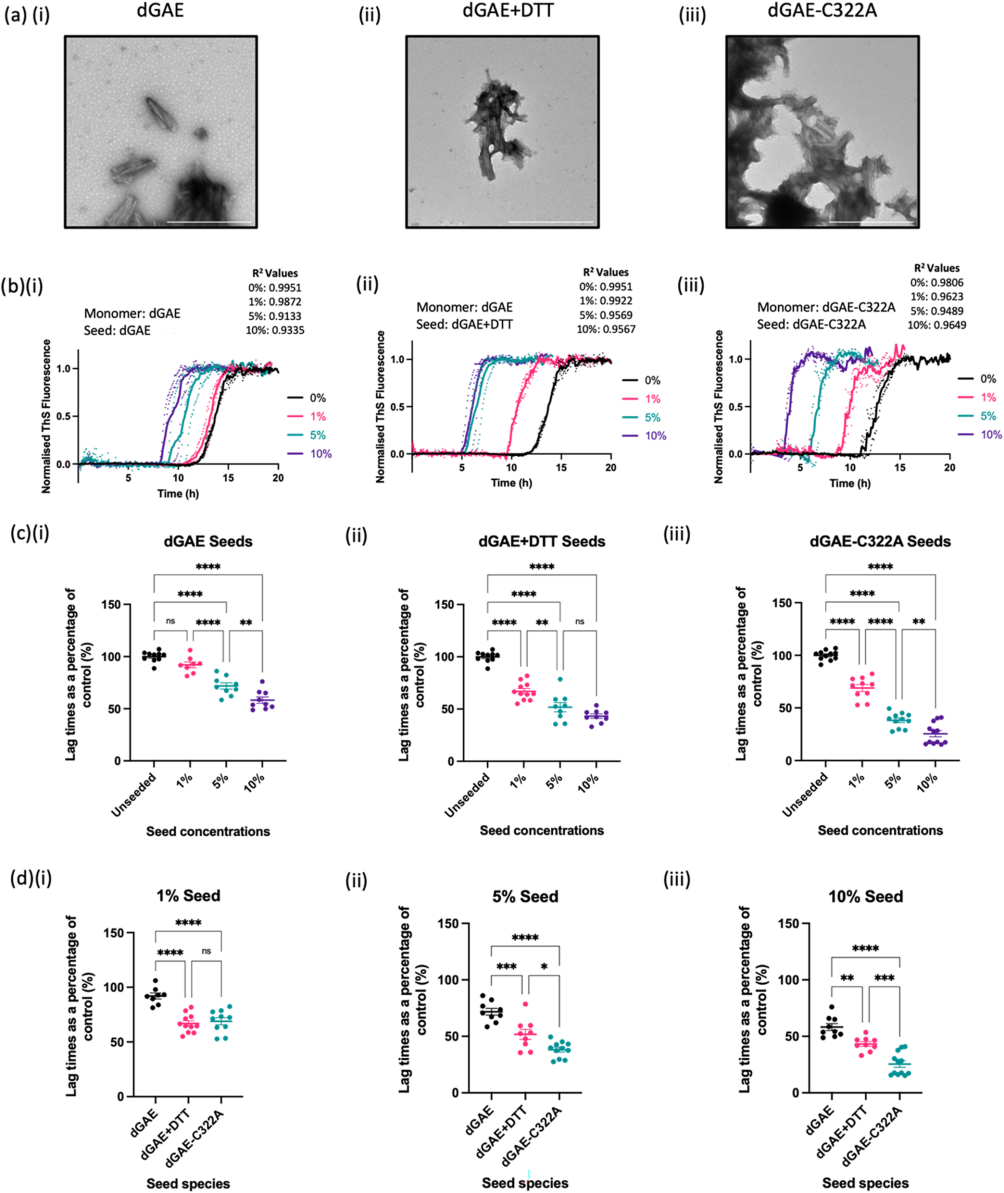
Columns show results arising from (i) dGAE assembled in nonreducing conditions, (ii) dGAE assembled in reducing conditions with 10 mM DTT, and (iii) dGAE-C322A assembled in nonreducing conditions. (a) Electron micrographs of seeds produced from sonicating fibrils produced in each condition. Scale bar represents 500 nm. (b) Examples of normalized thioflavin-S kinetics from a single experiment showing the seeding capability of each condition seeded with sonicated assemblies shown respectively in (a) at 0% (control, black), 1% (pink), 5% (green), and 10% (purple). Curves represent the mean values for each condition, with the *R* ^2^ value showing the goodness of fit for each curve. (c) Quantification and comparison of the lag times as a percentage of the unseeded control with the addition of 1, 5 and 10% of seeds from each condition. (c) (i) Seeds produced from dGAE in nonreducing conditions added to 10 μM of dGAE with 10 mM DTT. One-way ANOVA shows significant difference between groups (*F* = 54.41, *R* ^2^=0.8361, *p* < 0.0001) from 4 independent tests. Tukey’s multiple comparison test shows 1% dGAE seed (92.07 ± 2.750%) does not induce a significant reduction in lag phase when compared to the control (100 ± 1.608%, *p* < 0.0001), whereas the addition of 5% (71.77 ± 3.001%) and 10% (58.24 ± 3.023%) dGAE seeds does induce a significant reduction in lag phase when compared to the control (*p* < 0.0001). Significant reduction in the lag phase is also seen with the addition of 5% when compared to 1% dGAE seeds (*p* < 0.0001), and when comparing 10 to 5% dGAE seeds (*p* = 0.0047). (c) (ii) Seeds produced from dGAE in reducing conditions (10 mM DTT) added to 10 μM of dGAE with 10 mM DTT. One-way ANOVA shows significant difference between groups (*F* = 77.92, *R* ^2^ = 0.8698, *p* < 0.0001) from 4 independent tests. Tukey’s multiple comparison test shows 1% (66.97 ± 2.557%), 5% (51.76 ± 4.479%), and 10% (43.20 ± 2.069%) dGAE+DTT seed does induce a significant reduction in lag phase when compared to the control (100 ± 1.608%, *p* < 0.0001). Significant reduction in the lag phase is also seen with the addition of 5% when compared to 1% dGAE seeds (*p* = 0.0026), but not between 10 and 5% dGAE seeds (*p* = 0.1847). (c) (iii) Seeds produced from dGAE-C322A in nonreducing conditions added to 10 μM of dGAE-C322A. One-way ANOVA shows significant difference between groups (*F* = 179.6, *R* ^2^ = 0.9309, *p* < 0.0001) from 4 independent tests. Tukey’s multiple comparison test shows 1% (68.92 ± 3.247%), 5% (38.25 ± 2.127%), and 10% (25.47 ± 2.920%) dGAE+DTT seed does induce a significant reduction in lag phase when compared to the control (100 ± 1.399%, *p* < 0.0001). Significant reduction in the lag phase is also seen with the addition of 5% seed when compared to 1% dGAE seeds (*p* < 0.0001), and between 10 and 5% dGAE seeds (*p* = 0.0038). (d) Comparison of percentage lag times induced with the seeds produced from the different conditions at 1% (d)­(i), 5% (d)­(ii), and 10% (d)­(iii) using the same data from (c). (d) (i) One-way ANOVA shows significant difference between groups for 1% seed addition (*F* = 36.20, *R* ^2^ = 0.7283, *p* < 0.0001) from 4 independent tests. Tukey’s multiple comparison test shows a significant reduction in lag phase with the addition of dGAE+DTT and dGAE-C322A when compared to dGAE (*p* < 0.0001), but no significant difference observed between dGAE+DTT and dGAE-C322A seeds (*p* = 0.8734). (d) (ii) One-way ANOVA shows significant difference between groups for 5% seed addition (*F* = 36.20, *R* ^2^ = 0.7283, *p* < 0.0001) from 4 independent tests. Tukey’s multiple comparison test shows a significant reduction in lag phase with the addition of dGAE+DTT and dGAE-C322A when compared to dGAE (*p* < 0.0001), as well as significant difference observed between dGAE+DTT and dGAE-C322A seeds (*p* = 0.0153). (d) (iii) One-way ANOVA shows significant difference between groups for 10% seed addition (*F* = 36.20, *R* ^2^ = 0.7283, *p* < 0.0001) from 4 independent tests. Tukey’s multiple comparison test shows a significant reduction in lag phase with the addition of dGAE+DTT when compared to dGAE (*p* = 0.0033), a significant reduction with the addition of dGAE-C322A when compared to dGAE (*p* < 0.0001), as well as significant difference observed between dGAE+DTT and dGAE-C322A seeds (*p* = 0.0003).]

The rate constants obtained from the global fitted models reveal a substantial increase in the rate of the secondary process when DSB formation is inhibited either through addition of DTT or with the dGAE-C322A mutation (Supporting Information Figure S2a,b), and this is likely to be predominantly responsible for the faster assembly observed when DSB is inhibited. The rate of primary nucleation is also affected, although in different ways. While the C322A mutation resulted in a slight increase in the rate of primary nucleation, addition of DTT leads to a significant decrease in the rate of primary nucleation (Supporting Information Figure S2c). Secondary processes dominate in all cases, so the effect of primary nucleation on the overall speed of the assembly is less pronounced (Supporting Information Figure S2d). However, the decreased importance of primary nucleation upon DTT addition is evident in the assembly curves becoming sharper, with a more sudden increase after a flat lag phase.

TEM was carried out to observe the morphology of fibrils formed under different conditions at 15 μM. dGAE in nonreducing conditions formed short fibrillar species prone to lateral association (Figure 1d)­(i). In contrast, a higher proportion of elongated fibrils was observed in the reducing conditions (Figure 1d)­(ii) or using dGAE-C322A (Figure 1d)­(iii), and this is consistent with previous work.^19^ A direct comparison of ThS fluorescence traces from 15 μM of monomeric dGAE with or without DSB (Figure 1e) shows a significantly shorter lag phase of ∼11–12 h when DSB is inhibited (dGAE+DTT or dGAE-C322A) and ∼34 h for nonreduced dGAE (Figure 1f). Protein concentration in the supernatant following assembly was analyzed using a BCA assay (Supporting Information Figure 3). This gives a measure of unassembled/soluble species remaining the supernatant at time points during incubation and shows a gradual decrease in soluble protein with a saturation end point of approximately 38% for dGAE and 16% for dGAE+DTT at 168 h incubation. Interestingly, dGAE+DTT soluble concentration reduces rapidly after 2 h incubation (approximately 72%), while the rate of reduction is slower for dGAE. These data support the observations from ThS kinetics and suggest an increased efficiency of assembly for dGAE+DTT.

These mechanistic investigations illustrate that the assembly of dGAE is governed by fibril-dependent secondary processes, such as complex secondary nucleation processes on the surface of fibrils in solution through fragmentation or both. Although the overall mechanism is independent of DSB, the inhibition of these DSB leads to an enhanced assembly reaction with a significant reduction in the lag phase of assembly.

### dGAE Species Formed through the Inhibition of DSB Are More Capable of Seeding the Assembly of dGAE

Monitoring the assembly of proteins in the presence of preformed fibrils enables further examination of the involvement of secondary processes (secondary nucleation and fragmentation) by bypassing primary nucleation.^10^ Furthermore, it provides information on the differences in heterogeneous seeding capabilities between assemblies formed with DSB (DSB­(+)) in the dGAE sample, or prevention of DSB (DSB(−)) in the dGAE+DTT and dGAE-C322A samples.

The seeds were formed through previously optimized conditions and methods,^19,21,25^ to mimic the conditions used in recent cryo-EM studies using dGAE.^23^ dGAE (400 μM), with or without DTT (10 mM), or dGAE-C322A (400 μM) were agitated in an Eppendorf ThermoMixer at 400 rpm at 37 °C for 4d. Assemblies were isolated, and their concentrations were estimated, as described in Materials and Methods. The fibrils were sonicated before being added at 1, 5 and 10% of the initial monomeric protein concentration, 10 μM. Following sonication, TEM images showed short fibrils with no observable differences in morphology between the conditions (Figure 2a). dGAE and dGAE+DTT seeds were added to wild-type dGAE monomer in a reducing environment (10 mM DTT) because these conditions resulted in more reproducible ThS kinetic traces. dGAE-C322A seeds were added to dGAE-C322A monomer in nonreducing conditions.

The addition of 1% dGAE seeds to monomeric dGAE resulted in no significant reduction in the lag phase, whereas the addition of 5 and 10% seeds resulted in a significant reduction when compared to the unseeded control (Figure 2b­(i),c­(i)). A significant reduction in lag phase was observed when dGAE+DTT seeds (Figure 2b­(ii),c­(ii)) or dGAE-C322A seeds (Figure 2b­(iii),c­(iii)) were added at all percentages. These data show a clear reduction in the aggregation lag phase with the addition of preformed aggregates, providing clear evidence for the involvement of secondary nucleation in dGAE and dGAE-C322A assembly. The reduction in the lag phase is generally significantly concentration-dependent (Figure 2b,c). Although the reduction in lag phase for the increase from 5 to 10% dGAE+DTT was not significant.

Further analysis compared the normalized data as a percentage of the unseeded control (Figure 2d). The addition of 1% seed dGAE+DTT and dGAE-C322A seeds resulted in a significant reduction in lag phase when compared to the addition of dGAE seeds (Figure 2d­(i)). 5 and 10% seeds resulted in a significant difference between the three groups, whereby dGAE-C322A seeds significantly shorten the lag phase the most and dGAE-DTT the least (Figure 2d­(ii),d­(iii)). To summarize, these data showed that DSB(−) dGAE species (dGAE+DTT and dGAE-C322A seeds) were significantly more effective at seeding *in vitro* dGAE assembly when compared to DSB­(+) dGAE species. C322A dGAE appears to be the most effective seed, and this may be due to the complete removal of DSB in this sample.

Furthermore, the data also demonstrate that the introduction of sonicated, fibrillar dGAE species induced a reduction in the assembly lag phase, but it did not eliminate the lag phase completely, even at 10% of monomeric concentration. This is consistent with the view that surface-mediated nucleation dominates the seeding reactions,^26^ which supports the involvement of multistep secondary nucleation (Figure 1b). Additional seeding experiments showed that dGAE fibrils after sonication to produce smaller truncated fibrils are more competent seeds when compared to long mature fibrils (Supporting Information Figure S4). This could be evidence for the involvement of fragmentation in dGAE assembly, because of the increased presence of exposed fibril ends, facilitating the repeated cycles of elongation and fragmentation. Alternatively, this could be due to sonication helping to reduce clumping in the sample that facilitates the more competent seeding observed in sonicated samples, and not the length of the fibrils being the important factor.

The incomplete reduction in the lag phase with the addition of preformed seeds confirms the presence of secondary processes in the assembly of dGAE at all conditions. More importantly, there was a difference in the ability of the seeds to accelerate the assembly of dGAE, when comparing seeds formed in conditions favoring DSB and with those preventing DSB. Our results suggest that DSB(−) dGAE seeds are more capable of seeding the *in vitro* assembly of dGAE.

### The Disulfide Bond Influences the Macromolecular Structure of the dGAE Filaments

Cryo-EM revealing the structurally distinct tau filaments seen in tauopathies has highlighted the relationship between amyloid filament polymorphic structures and disease.^5,6,8^ Next, we focused on investigating the differences in the structure of dGAE filaments formed in conditions favoring and inhibiting DSB to gain insights into the effect of conditions on polymorphs.

To investigate the differences in species present during the assembly of dGAE in the different conditions, whole assembly samples were taken at 0, 2, 4, 6, 24, 72, and 7 days aggregation and run on a nonreducing gel to identify the SDS-soluble species present. We have previously reported that dGAE monomer migrates on a gel as a doublet at 10 and 12 kDa, which are prone to dimerize, forming dimers at 20 and 24 kDa, respectively.^19^ The dGAE mixture clearly shows these species, with a strong presence of monomers and dimers throughout the assembly, with a stronger presence of the 12 kDa monomer and its 24 kDa dimer, when compared to the 10 kDa monomer and 20 kDa dimer (Figure 3a­(i)). Upon 24 h, there is the presence of higher molecular species with faint bands at ∼50 and ∼125 kDa, which is to be expected with the formation of fibrillar species. However, with the inhibition of the DSB with DTT (Figure 3a­(ii)) and the C322A variant (Figure 3a­(iii)), there is no dimer present at 0 h, with the introduction of a faint band at 24 kDa from 4 h that gets stronger throughout assembly due to the formation of larger species that are SDS-soluble. The similarity between the dGAE+DTT and dGAE-C322A results shows that this dimer must be disulfide-independent and cannot be due to a reduction in the effect of the DTT in the reduced sample. It would be assumed that the strong presence of a dimer in the dGAE sample would reduce the presence of the monomer in the sample, but this is not easily seen with this analysis. These gels indicate a clear difference in species present between assembly conditions and allude to the formation of fibrils that exhibit structural differences, due to the variation in their SDS solubility.

**Figure 3:**
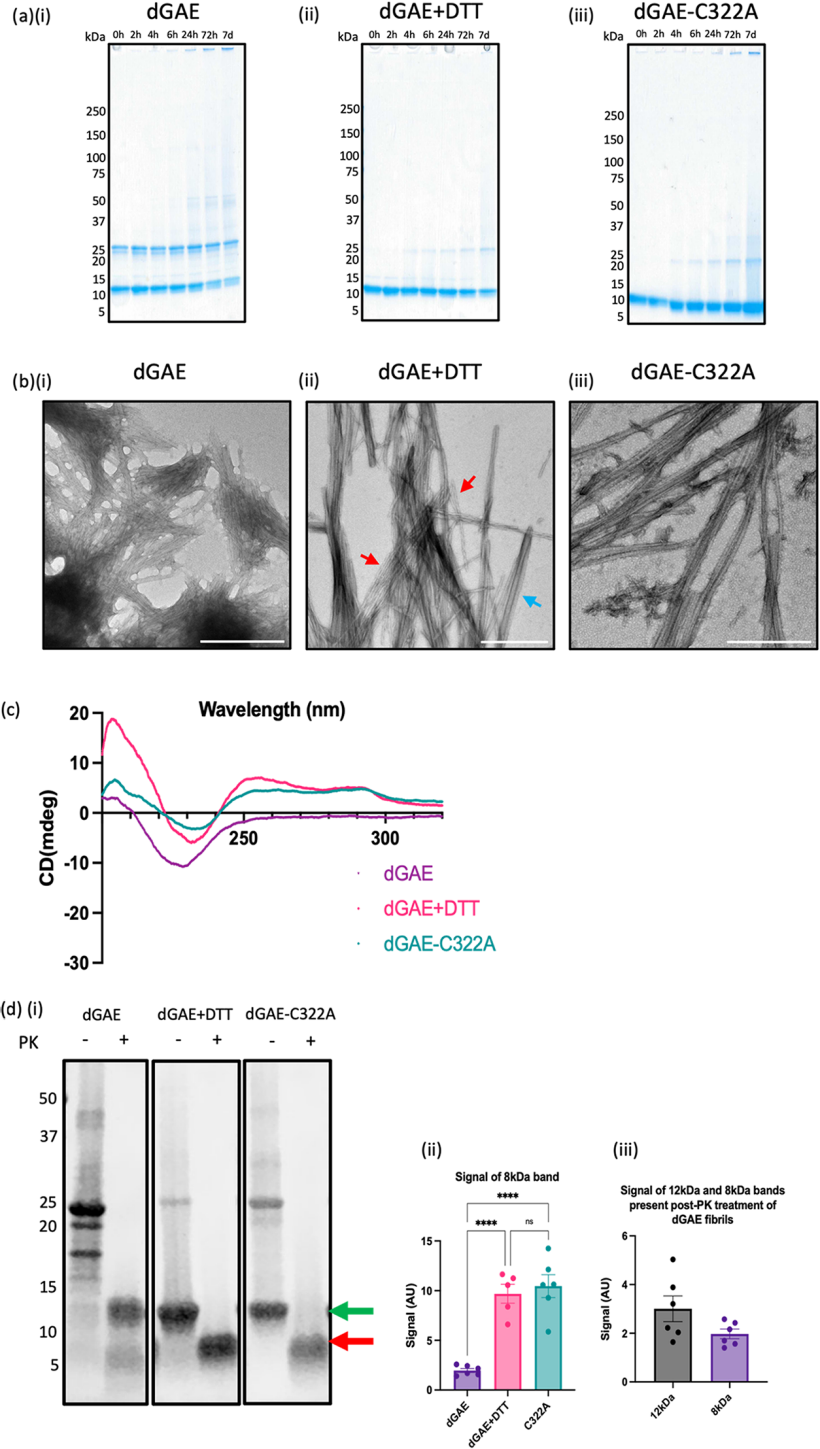
Macromolecular differences between fibrils assembled in conditions that favor disulfide bonding compared to fibrils assembled in conditions inhibiting disulfide bonding. dGAE (± 10 mM DTT) and dGAE-C322A assembled over 7 days, with samples of the assembly mixture removed at time points throughout assembly at 0 h, 2 h, 4 h, 6 h, 24 h, 72 h, and 7 days. The samples of the whole assembly mixture (aggregated and soluble protein) were mixed with Laemmli sample buffer without BME and loaded onto an Any kDa Mini-PROTEAN precast gel to isolate the SDS-soluble species present at each time point of assembly in the three conditions dGAE (nonreduced: a­(i)), dGAE+DTT (reduced: a­(ii)), and dGAE-C322A (a­(iii)). Electron micrographs of fibrils formed from 400 μM dGAE in nonreducing conditions (b­(i)) and in reducing conditions with 10 mM DTT (b­(ii)) and 400 μM dGAE-C322A in nonreducing conditions (b­(iii)). Scale bar represents 500 nm. (c) CD spectrum from the pellet from each condition. (d­(i)) SDS-PAGE and Coomassie staining for 9 μL of 200 μM isolated fibrils only from each condition with and without proteinase K treatment. Arrows represent the difference between the 12 kDa (green) and 8 kDa (red) MW bands. (d­(ii)) Quantification and comparison of the signal from the 8 kDa band for each condition after proteinase K treatment. [One-way ANOVA showed significant difference between each group (*F* = 30.64, *R* ^2^ = 0.8140, *p* < 0.0001) from 5 independent tests. Holm-Šídák’s multiple comparisons test showed a significant increase in the signal from the 8 kDa band in the dGAE+DTT sample (9.686 ± 0.9527) and dGAE-C332A sample (10.45 ± 1.150) when compared to the dGAE sample (1.973 ± 0.1982, *p* < 0.0001). There is no significant difference between dGAE+DTT and dGAE-C322A. (d­(iii)) Quantification and comparison of the signal from the 12 and 8 kDa MW band from the dGAE sample. Unpaired *t* test showed no significant difference between each group (*F* = 7.072, *R* ^2^ = 0.2516, *p* = 0.0966) from 6 independent tests].

Lower starting monomer concentrations (∼15 μM) showed that prevention of DSB resulted in longer fibrils (Figure 1d­(ii),(iii)). At 400 μM, electron micrographs show that reducing conditions result in filaments that are longer than those formed in nonreducing conditions (Figure 3b­(i),(ii)). The reducing conditions result in a mixture of twisted filaments that mimic PHF (red arrows),^21,23^ and straight filaments that laterally associate to form thicker filaments (blue arrows). dGAE-C322A monomer assembles to form straight laterally associated filaments that resemble the filaments seen in the dGAE+DTT sample shown with the blue arrows, but PHF-like twisted filaments were not observed (Figure 3b­(iii)). A higher starting monomer concentration (400 μM) resulted in samples that contained mostly elongated filaments (Figure 3a­(i)–(iii)), whereas filaments formed using low concentrations (15 μM) withdrawn from kinetics assays were overall shorter and contained less elongated fibrils (Figure 1d­(i)–(iii)).

The CD spectra of dGAE filaments after the fibrils have been isolated through centrifugation, showed a minimum at ∼225 nm and a maximum at ∼200 nm, arising from a high β-sheet content as expected for fibrils (Figure 3c).^19^ The DSB(−) fibrils also show this strong β-sheet signal, but with two additional maxima at ∼250 nm and ∼290 nm, which suggests an increased order for these filaments compared to the dGAE filaments that may arise from stacking of tyrosine residues.^27^ The high tension output during CD data collection can provide information about absorbance from additives. Measurements taken during CD of samples with and without DTT show little difference in signals, suggesting the washing stages done when preparing the dGAE+DTT fibrils remove the excess DTT and remove the interference associated with DTT in CD measurements (Supporting Information Figure S5).

Structural variation between DSB­(+) and DSB(−) filaments was further investigated by examining surface exposure, using protease resistance to Proteinase K (PK) digestion.^28^ Fibrils formed from dGAE, dGAE+DTT, and dGAE-C322A at 400 μM were diluted to 200 μM and incubated with 25 μg/mL PK for 1 h at 37 °C, and the products were examined using SDS-PAGE (Figure 3d­(i)). Without PK digestion, there is a noticeable difference in the SDS-soluble species between conditions. dGAE filaments are SDS-soluble and mostly run as apparent dimers, shown at 20 and 24 kDa, corresponding to their 10 and 12 kDa monomers shown in our previous work.^19^ We also see a minor band at a higher molecular weight band around 40 kDa and another lower at ∼17 kDa that is not seen for the dGAE+DTT or dGAE-C322A fibril samples. dGAE+DTT and dGAE-C322A run with a far less intense band at 24 kDa (non-DSB dimer) and a stronger band at 12 kDa, representing monomeric dGAE (green arrow). The very weak 24 kDa band present in the dGAE+DTT and dGAE-C322A samples must be disulfide-independent dimers. Post-PK digestion, dGAE filaments formed in nonreducing conditions run at 8 kDa (red arrow), showing a truncated dGAE monomer consisting of the protease-resistant core, and another band at a molecular weight of 12 kDa, which could represent the full-length dGAE monomer (12 kDa). Mass spectrometry of the 8 kDa band showed that this fragment maps to a protease-resistant core of dGAE filaments encompassing the region His299–Lys370 (Figure S6a). These two distinct bands post-PK treatment were observed even with an increase in PK concentration up to 250 μg/mL, suggesting that their presence was not due to incomplete digestion (Figure S6b). dGAE+DTT and dGAE-C322A fibrils run with a single band at 8 kDa, which is significantly stronger when compared to the dGAE fibrils (Figure 3d­(ii)). Overall, the PK digestion data highlight differences in the structural cores of the fibrils formed from DSB(−) and DSB­(+) dGAE fibrils.

These observations may suggest that the dGAE sample has two populations of filaments, one with DSB and one without DSB. The latter population would show filaments having characteristics similar to those formed in the dGAE+DTT and dGAE-C322A samples, where DSB is inhibited. The filaments can be partially digested by PK before being broken down into monomers by SDS, which yields the band at 8 kDa. The other population (12 kDa) consists of a filament structure that is affected sufficiently by the PK for it to become more SDS-soluble since there is no 12 kDa band without PK, but a strong 12 kDa band with PK. Quantification of the 12 vs 8 kDa band of dGAE fibrils with PK showed us that there is generally more of the 12 kDa band than the 8 kDa band. This difference was not significant but demonstrates that there is a greater presence of the fibrils giving rise to 12 kDa band than those responsible for the 8 kDa band (Figure 3d­(iii)).

These data suggest that DSB has a significant effect on the macromolecular structure of dGAE filaments, which could explain the differences in assembly kinetics and seeding propensity observed previously. DSB(−) fibrils show a distinct CD spectrum, SDS-PAGE profile, and susceptibility to protease digestion when compared with DSB­(+) fibrils.

### DSB­(−) Species Can Recruit Endogenous Tau in Tau Biosensor Cells

Having shown that DSB(−) dGAE species are more capable of seeding assembly compared with DSB­(+) dGAE species *in vitro*, we investigated whether dGAE aggregates are able to recruit endogenous tau within cells. We utilized the Tau RD P301S FRET Biosensor model of tau aggregation (tau Biosensor cells), in which HEK293T cells express two populations of tau corresponding to the repeat domain (RD) region and carrying the P301S mutation associated with FTD.^24^ Each population of tau has a separate fluorescent tag, either CFP or YFP. The addition of seed-competent tau leads to the aggregation of endogenous tau, and the resultant close proximity of the two populations of tau results in fluorescent puncta, which can be interpreted as the aggregation of endogenous tau.

We utilized the automated ImageXpress Pico system to develop a highly sensitive method of measuring and analyzing the fluorescence signals in live tau Biosensor cells (Supporting Information Figure S7). Cells were plated in a 96-well plate and treated with soluble dGAE or dGAE-C322A, and sonicated or nonsonicated fibrils (Figures 2a­(i–iii) and 3b­(i–iii), respectively). The addition of nonaggregated soluble dGAE protein resulted in no appreciable fluorescent signal intensity, suggesting that soluble dGAE and dGAE-C322A did not recruit endogenous tau to assemble during the time-course of the experiment (Figure 4a,b­(i)). DSB­(+) dGAE fibrils and sonicated fibrils did not induce a fluorescent signal, suggesting that the DSB­(+)­species were unable to recruit endogenous tau. However, there was a significant increase in the fluorescence puncta with the incubation of dGAE+DTT and dGAE-C322A fibrils (DSB(−)) and sonicated fibrils compared to the DSB­(+) dGAE fibrils and sonicated fibrils (Figure 4a,b). This suggests that only DSB(−) fibrils can recruit cellular endogenous tau to form aggregates.

**Figure 4:**
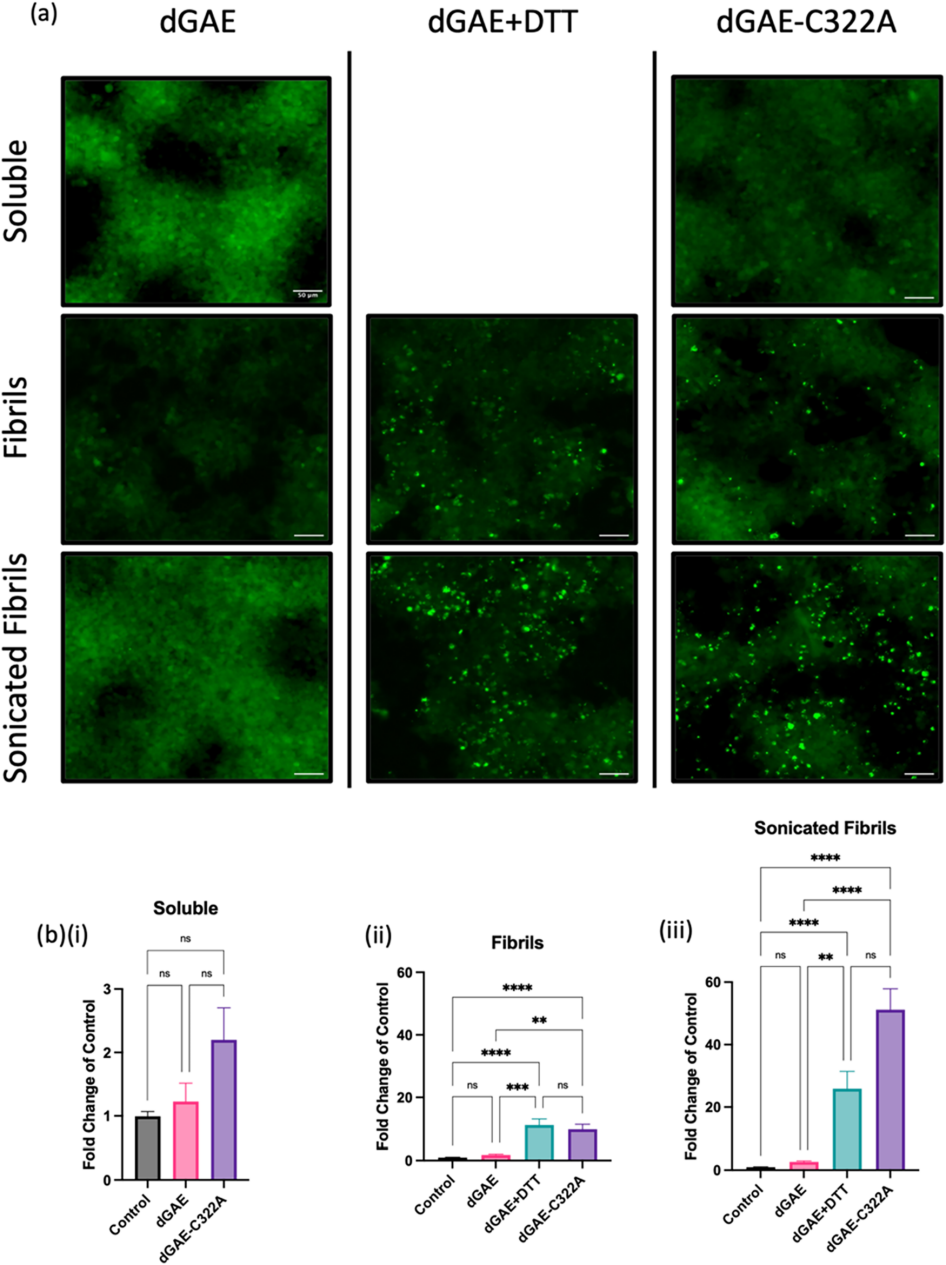
Tau biosensor cells were incubated with 10 μM of each of the dGAE species for 3 days before imaging. (a) Representative images of tau Biosensor cells taken from each condition using an ImageXpress Pico. The fluorescent signal appears as a punctate green fluorescence and diffuse green signal seen in cells without a punctate signal. Scale bars represent 50 μm. Data are not shown for soluble dGAE-DTT. (b) Quantification and comparison from analysis of fluorescence in response to soluble dGAE and dGAE-C322A when compared to the control. [Kruskal–Wallis test showed no significant difference between samples (*p* = 0.1396).] (c) Quantification and comparison from analysis of fluorescence induced by dGAE, dGAE+DTT, and dGAE-C322A fibrils when compared to the control. [Kruskal–Wallis test showed no significant difference between samples (*p* < 0.0001). Dunn’s Multiple comparison test showed dGAE-C322A fibrils (9.825 ± 1.60) induced a significant increase in fluorescence when compared to control (1.00 ± 0.07265, *p* < 0.0001) and dGAE fibrils (1.795 ± 0.2744, *p* = 0.0012). dGAE+DTT (11.19 ± 1.917) fibrils induce a significant increase in fluorescence compared to control (*p* < 0.0001) and dGAE fibrils (*p* = 0.0003). No significant difference between dGAE+DTT and dGAE-C322A fibrils. (d) Quantification and comparison from the analysis of fluorescence induced by dGAE, dGAE+DTT, and dGAE-C322A sonicated fibrils when compared to control. Kruskal–Wallis test showed no significant difference between samples (*p* < 0.0001). Dunn’s multiple comparison test showed dGAE-C322A fibrils (51.15 ± 6.726) induced a significant increase in fluorescent puncta when compared to control (1.00 ± 0.07265, *p* < 0.0001) and dGAE fibril (2.689 ± 0.2828, *p* < 0.0001). dGAE+DTT (25.86 ± 5.554) fibrils induced a significant increase in fluorescent puncta compared to control (*p* < 0.0001) and dGAE fibrils (*p* = 0.0092). There was no significant difference between seeding with dGAE+DTT and dGAE-C322A fibrils.]­dGAE+DTT and dGAE-C322A induce signals in tau biosensor cells.

The above findings provide further evidence of distinct differences in the macromolecular structure of fibrils formed in the absence of DSBs that facilitate their ability to seed aggregation. The addition of sonicated fibrils caused a significant increase in the fluorescent signal compared to nonsonicated fibrils, potentially because of their smaller size making it easier for their internalization within the cell.

## Discussion

In this study, we aimed to evaluate the self-assembly mechanisms involved in dGAE amyloid formation and the contributions of DSB to these mechanisms, filament structure, and role in tau pathology. We first optimized conditions for reproducible self-assembly of the dGAE tau fragment in environments that either favor DSB or inhibit DSB (using reducing conditions with 10 mM DTT and a cysteine variant of dGAE). ThS fluorescence assays from a monomeric solution showed that dGAE aggregation demonstrated the expected sigmoidal curve with a pronounced lag time, followed by a sudden elongation phase and a reaction order close to −0.5, which suggests that secondary processes (secondary nucleation and fragmentation) dominate the assembly process. The dGAE aggregation kinetics fitted closely with the aggregation models of multistep secondary nucleation and saturating elongation with fragmentation (Figure 1), and the seeding assays showed a clear accelerated assembly with the addition of aggregates (Figure 2). The reduction, but not the elimination, of the lag phase is evidence for a surface-mediated seeding process (multistep nucleation). Surface-mediated assembly is still a nucleation-dependent process, requiring a slow nucleation phase, albeit an enhanced reaction when compared with the absence of seeds. This has been reported for Aβ42 monomer and seeds produced from the yeast prion-forming protein, Sup35NM.^26^ Experimentally, we also show that sonicated fibrils were more competent seeds when compared to fibrils (Figure S4). We predicted that a seed containing a larger fibril surface, such as long fibrils, should facilitate the multistep secondary nucleation process that occurs on the fibril surface, whereas a seed with more exposed ends, such as sonicated fibrils, should facilitate the elongation with fragmentation process that occurs at the ends of fibrils. This suggests that the sonicated fibrils, with more exposed ends, favored the elongation and fragmentation processes, suggesting the presence of fragmentation in dGAE aggregation. However, it is difficult to determine whether the sonication aided seeding due to the number of seeds or aiding their availability by influencing their size. Nevertheless, it is clear that sonication has a significant effect on increasing the seeding ability of dGAE-C322A seeds *in vitro*, which is later also shown in tau Biosensor cells. It is also important to consider the involvement of agitation in our experimental conditions, since we were unable to obtain reproducible spontaneous assembly of dGAE without agitation. The agitation of the sample to induce reproducible aggregation kinetics may cause fragmentation of aggregated species.^29^ Considering all the evidence we have gathered for dGAE aggregation in the absence and presence of seeds, we suggest that both elongation with fragmentation and multistep secondary nucleation are active in the process of dGAE aggregation. These processes are not mutually exclusive, and it is important to highlight their presence within a complex assembly process that makes it difficult to draw a conclusion about the precise mechanism involved.

Investigating the self-assembly of amyloidogenic proteins under varying conditions helps to highlight important mechanisms during the assembly process and how that translates to the formation of pathological aggregates found in the brain. DSB has been a debated topic for tau assembly, with studies using previous models of *in vitro* tau aggregation (T40 and K18/K19) suggesting that DSB is essential for self-assembly,^17^ whereas our previous work using dGAE has shown that inhibition of DSB enhances self-assembly.^19^ This study has provided a more detailed mechanistic investigation, using ThS aggregation traces, to study the impact of inhibiting DSB. Our results show that the inclusion or inhibition of DSB has little effect on the global mechanism of assembly for dGAE, with secondary nucleation processes dominating the assembly reaction (Figures 1 and S2). However, there is a clear acceleration of aggregation through a significant reduction in the lag phase when DSB is inhibited. This suggests that DSB does not affect the overall mechanism of self-assembly but the absence of DSB enhances the aggregation of dGAE and reduces the lag phase of assembly. This led us to investigate the ability of the species formed from each condition to act as a seed. We found that DSB(−) dGAE seeds are more seed competent than DSB­(+) species, in both *in vitro* aggregation (Figure 2) and in recruiting endogenous tau in the tau biosensor cells (Figure 4). This suggests that there are subtle differences in the structure of the fibrils produced under different conditions that result in a species with varying seeding capabilities to facilitate the secondary processes of assembly observed with our mechanistic studies. With these observations, we have devised a model to explain the enhanced assembly observed in the dGAE+DTT and dGAE-C322A samples and the increased ability of DSB(−) species at seeding aggregation (Figure 5). We propose that the inhibition of DSB during dGAE assembly produces dGAE aggregates that are more seed-competent, which accelerates further aggregation, while allowing the formation of DSB results in far fewer of these seed-competent species, which shows in a much slower assembly reaction. This is also supported by the finding that secondary processes are dominant within the assembly reaction, whether DSB is allowed or inhibited, meaning that these seed-competent species play a crucial role in dGAE assembly. PK digestion assays (Figure 3) also suggested that there are two populations of filaments with different proteolytic profiles, whereas only one population is found in the sample DSB- sample. SDS-PAGE analysis of the assembly mixture shows that in nonreduced conditions, there is a strong dimer presence throughout the assembly, which is absent in the reduced sample, indicating a disulfide dependence. These disulfide-dependent dimers may be seed-incompetent, which slows assembly and reduces monomer availability for aggregation, further slowing assembly. Although the reduction in monomer is not clear on the gel, it would be expected that such a strong presence of a dimer will reduce the monomer available for assembly. In contrast, the reduced and C322A samples only show a small non-DSB dimer presence, starting from 4 h of agitation. This also suggests that the dGAE+DTT sample is sufficiently reduced throughout the assembly. These gels clearly show a significant difference in the assembly mixtures between the nonreduced and reduced samples, further suggesting the inhibitory role of DSB in dGAE assembly. It is important to consider that these gels only highlight the presence of SDS-soluble species and do not give a clear insight into the species that make up the aggregated species.

**Figure 5:**
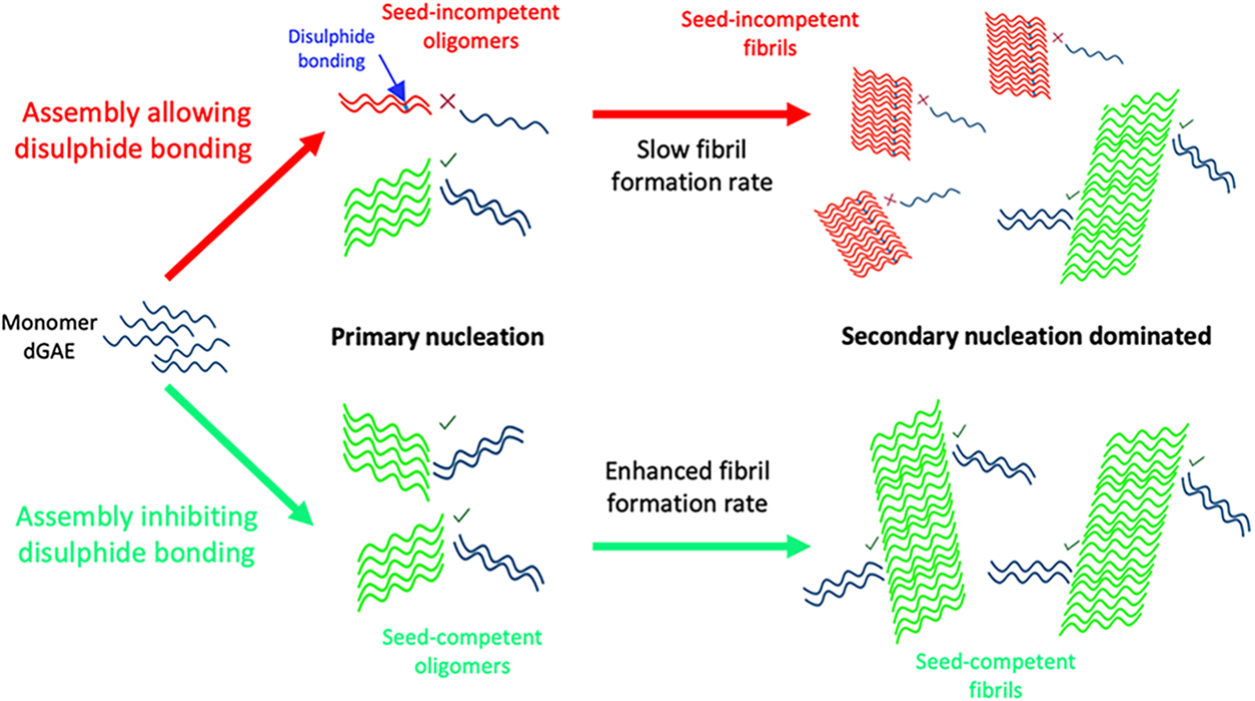
We speculate that the assembly in nonreducing conditions results in a mixture of oligomers after primary nucleation with and without disulfide bonding. The oligomers with disulfide bonds (illustrated in red with blue connections) are less seed incompetent, while the oligomers without disulfide bonds are more seed competent (shown in green). Due to fewer seed-competent oligomers, the secondary dominant process into mature fibrils is slow, resulting in a mixture of seed-incompetent and seed-competent fibrils. In DSB(−) conditions, disulfide bonds are inhibited, which leads to the primary nucleation of seed-competent oligomers only. This means that secondary nucleation is facilitated, and there is an enhanced fibril formation. This resulted in a sample of fully seed-competent fibrils. Created with permission from BioRender.com.

There is a significant difference in the lag phase of *in vitro* aggregation between dGAE+DTT and dGAE-C322A, and a slight (nonsignificant) increase in the seeding of endogenous tau in the tau Biosensor cells with the addition of the dGAE-C322A sonicated fibrils. This could signify that dGAE-C322A aggregates are superior at seeding when compared to dGAE+DTT, perhaps total removal of the ability to form DSB in the C322A variant. Alternatively, it may be due to discrepancies in the quantification of the fibril concentrations between the standard BCA used for the dGAE-C322A sample and the reducing agent-compatible BCA used for the dGAE+DTT sample.

Further differences in the structure of the fibrils were observed using TEM, CD, and SDS-PAGE and proteolysis analyses (Figure 3). CD results show two distinct spectra between the fibril types, with the fibrils formed in the absence of DSB containing two additional peaks at ∼250 and ∼290 nm, which may be due to stacking of tyrosine residues.^27^ SDS-PAGE illustrated differences in the presence of species in the DSB(−) and DSB­(+) aggregates after post-SDS degradation. Previously, we reported that dGAE fibrils formed under reducing and nonreducing conditions differed in terms of the extent of core using cross-polarization and INEPT solid-state NMR.^30^ Fibrils formed without DTT were more dynamic, suggesting that there was less of the protein incorporated into a stable core compared with the well-ordered, static core found in dGAE+DTT fibrils. In this study, PK treatment was used to further investigate differences in the structure and protease resistance of the fibrils. dGAE+DTT and dGAE-C322A fibrils exhibit one strong band at 8 kDa post-treatment. This is likely to be the partially digested dGAE monomer that forms a protease-resistant, but SDS-soluble, core of the fibrils formed in the absence of DSB. However, in the dGAE filaments, we see the presence of two distinct bands, one band at 8 kDa, representing the protease-resistant core and the other at 12 kDa. At first, this was assumed to be due to insufficient PK digestion, resulting in undigested protein. But the presence of these two bands persisted for PK concentrations up to 250 μg/mL, suggesting that this is not the case (Figure S6). We propose that the dGAE sample contains two separate populations of fibrils. One fibril population is formed without DSB, which is the dominant fibril seen in the dGAE+DTT and dGAE-C322A samples and is found as an 8 kDa band following PK digestion. The other fibril population is formed with DSB and has a conformation that is resistant to PK. Following electrophoresis, the 12 kDa band is present, but PK is still able to affect their conformation by making the filaments more SDS-soluble, shown by a lack of 20/24 kDa dimer post-PK treatment (Figure 6).

**Figure 6:**
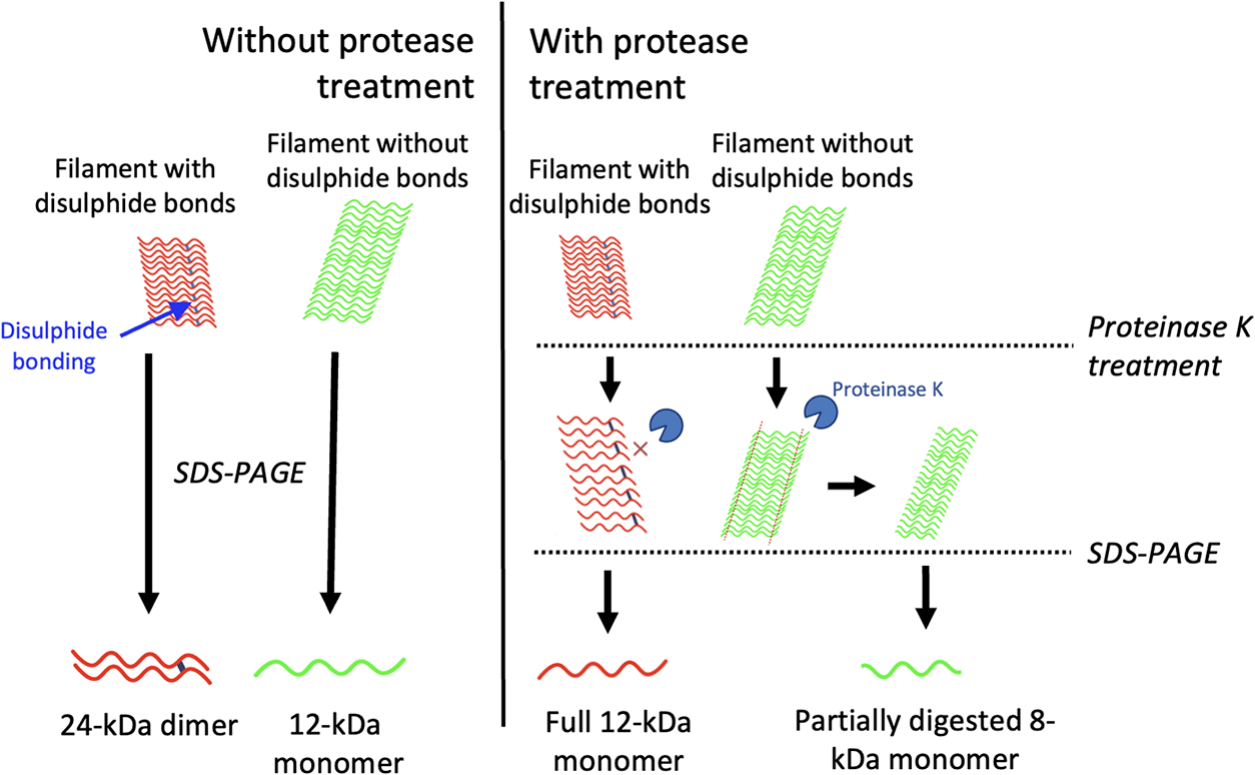
Filaments with disulfide bonds (red filaments with blue connections) are broken down mainly to dGAE dimers (24 kDa), seen with SDS-PAGE, whereas filaments without disulfide bonds (green filaments) are broken down to 12 kDa species after separation by SDS-PAGE. Proteinase K is able to partially digest filaments with disulfide bonds to generate a 12 kDa species. In contrast, filaments without disulfide bonds are less resistant to proteinase K activity, resulting in partially digested monomer, which are then seen as 8 kDa monomers with SDS-PAGE. Created with BioRender.com.

To gain further insights into the relevance of the seeding process for tau pathology, we utilized tau biosensor cells to investigate which species were able to seed the assembly of endogenously expressed forms of tau. Results revealed that only fibrillar, and not monomeric, forms were able to seed assembly and that sonicated fibrils were more efficient at seeding than nonsonicated fibrils. Importantly, it appeared that DSB­(+) dGAE species were unable to seed endogenous tau assembly, whether fibrillar or sonicated. In contrast, DSB(−) dGAE species were effective in the recruitment of endogenous tau in the tau Biosensor cells without the need for agents, such as Lipofectamine 2000, to aid internalization. Sonicating the fibrils resulted in a significant increase in punctate fluorescence, suggesting that the sonicated fibrils are superior at seeding the endogenous tau. This could be because smaller seeds are better at internalizing within the cell, or smaller dGAE species are better at seeding, which was observed *in vitro* (Supporting Information Figure S4). These results indicate that dGAE aggregates are able to enter cells and recruit endogenous tau, further highlighting the use of dGAE aggregates in the study of tau seeding and propagation. We avoided using transfection agents to aid internalization of aggregated species to reflect a more physiological uptake into the cells. Therefore, the varying effects observed between the samples could be due to variations in uptake of the species. However, we have previously reported that dGAE internalizes within the cell^31^ and believe the variation in seeding observed is due to the ability of the species to recruit endogenous tau to aggregate. The various profiles of species present after PK digestion might also suggest that DSB(−) dGAE aggregates are more protease-resistant and, therefore, are more likely to persist within the cell to recruit endogenous tau. This is consistent with a proteolytic selection process discussed by Bansal and colleagues, who state that fibril formation results in a mixture of polymorphic filaments that vary in their protease stability, and proteolytic activity leads to the degradation of soluble proteins, resulting in only protease-stable filaments.^28^ Our data suggest that tau fibrils formed with DSB could be more susceptible to digestion, whereas the DSB(−) fibrils are more protease-resistant and able to persist in the cell. These fibrils also exhibit the pathological characteristics of being more seed-competent, which we might expect to facilitate the recruitment of endogenous tau for the propagation of tau pathology throughout the brain.

Taken together, our data suggest that the inhibition of DSB is a key step in the self-assembly of dGAE and the production of pathological seed-competent species of tau. Identifying the inhibition of DSB as an essential pathological step in dGAE aggregation is consistent with studies investigating tau aggregates in disease. Cryo-EM structures of PHFs in AD revealed that the cysteine residues are buried deep within the structure of the filament and therefore unavailable for DSB.^5^ In addition, the predominantly reducing environment of the cell cytosol^32^ would favor the inhibition of DSB and the formation of these more seed-competent species that we have shown with dGAE+DTT and dGAE-C322A. We propose that the balance between the structures of the fibrils formed through DSB or by inhibiting DSB is responsible for the speed of aggregation of dGAE and the seeding capabilities of the aggregated dGAE species in vitro and in tau Biosensor cells.

In conclusion, we have utilized the dGAE fragment to investigate the role of DSB in tau self-assembly. We suggest that the inhibition of DSB is an important step toward pathological self-assembly of dGAE and the formation of aggregated tau species capable of propagating tau pathology. Our data indicate that this is due to a distinct difference in fibril structure as a result of the inhibition of DSB, which produces a more seed-competent dGAE species shown within in vitro assembly assays and within a tau aggregation cellular model. Recent cryo-EM studies have illustrated that subtle but distinct tau fibril polymorphs associated with tauopathies, highlighting the importance of structure and assembly environment in the study of tau pathology. Here, this has now been extended to the dGAE tau aggregation model, illustrating that varying the assembly conditions can have a drastic effect on the assembly and fibril characteristics. This further demonstrates the use of the dGAE in vitro model in investigating the mechanisms that play a pivotal role in the stages of aggregation and tau propagation and in the development of tau-targeted therapies.

## Supplementary Material



## Data Availability

The datasets supporting the conclusions of this article are included within the article and its supporting information.

## Notes

Fitting of global assembly mechanisms to ThS kinetics traces (Figure S1); primary and secondary rate constants of assembly from each condition (Figure S2); presence of monomeric dGAE throughout assembly in nonreduced and reduced conditions (Figure S3); effect of sonication on seeding ability of dGAE-C322A seeds (Figure S4); HT reading of each fibril during CD (Figure S5); basis for fibrillar protease K resistance (Figure S6); and raw images and analysis overlay of tau Biosensor cells acquired using ImageXpress Pico and analyzed in CellReporterXpress (Figure 7S) (PDF)

## Notes

If an author’s address is different from the one given in the affiliation line, this information may be included here

## Abbreviations

YT: yeast tryptone 2xYT
IPTG: isopropyl β-D-1-thiogalactopyranoside
EDTA: ethylenediaminetetraacetic acid
DSB: disulfide bonding
DTT: dithiothreitol
EGTA: ethylene glycol-bis­(2-aminoethyl ether)-*N*,*N*,*N*′,*N*′-tetraacetic acid
PB: phosphate buffer
ThS/ThT: thioflavin S/T
BCA: bicinchoninic acid
TEM: transmission electron microscopy
CD: circular dichroism
PK: proteinase K
SDS-PAGE: sodium dodecyl-polyacrylamide gel electrophoresis
