# Supplementary material for: Inhibiting Disulfide Bonding in Truncated Tau297–391 Results in Enhanced Self-Assembly of Tau into Seed-Competent Assemblies
Source: ACS Chem Neurosci. 2025 Dec 10;17(1):124–38. doi: 10.1021/acschemneuro.5c00639 (PMC12784331; doi:10.1021/acschemneuro.5c00639)
Supplement: Supplementary file 1 [file cn5c00639_si_001.pdf]

Supplementary information

Inhibiting disulphide bonding in truncated tau297-391 results in enhanced self-assembly of tau into seed-competent assemblies.

Sebastian S. Oakley[1, 2], Karen E. Marshall[1, 2], Georg Meisl[3], Alice Copsey[1], Mahmoud B. Maina[1, 2], Robert Milton[4], Thomas Vorley[4], John M. D. Storey[5, 6], Charles R. Harrington[4, 6], Claude M. Wischik[4, 6], Wei-Feng Xue[7] and Louise C. Serpell[1, 2*]

[1] Sussex Neuroscience, School of Life Sciences, University of Sussex, Falmer, East Sussex, BN1 9QG, UK

[2] Biomedical Science Research and Training Centre, Yobe State University, Damaturu, Yobe State, Nigeria

[3] Yusuf Hamied Department of Chemistry, University of Cambridge, Lensfield Road, Cambridge, Cambs, CB2 1EW, UK

[4] Institute of Medicine, Medical Sciences and Nutrition, University of Aberdeen, Aberdeen, AB24 3FX, UK

[5] Department of Chemistry, University of Aberdeen, Aberdeen, AB24 3FX UK

[6] TauRx Therapeutics Ltd., 395 King street, Aberdeen, AB24 5RP UK

[7] School of Bioscience, Division of Natural Science, University of Kent, Canterbury, CT2 7NZ, UK

Supplementary Figure 1.

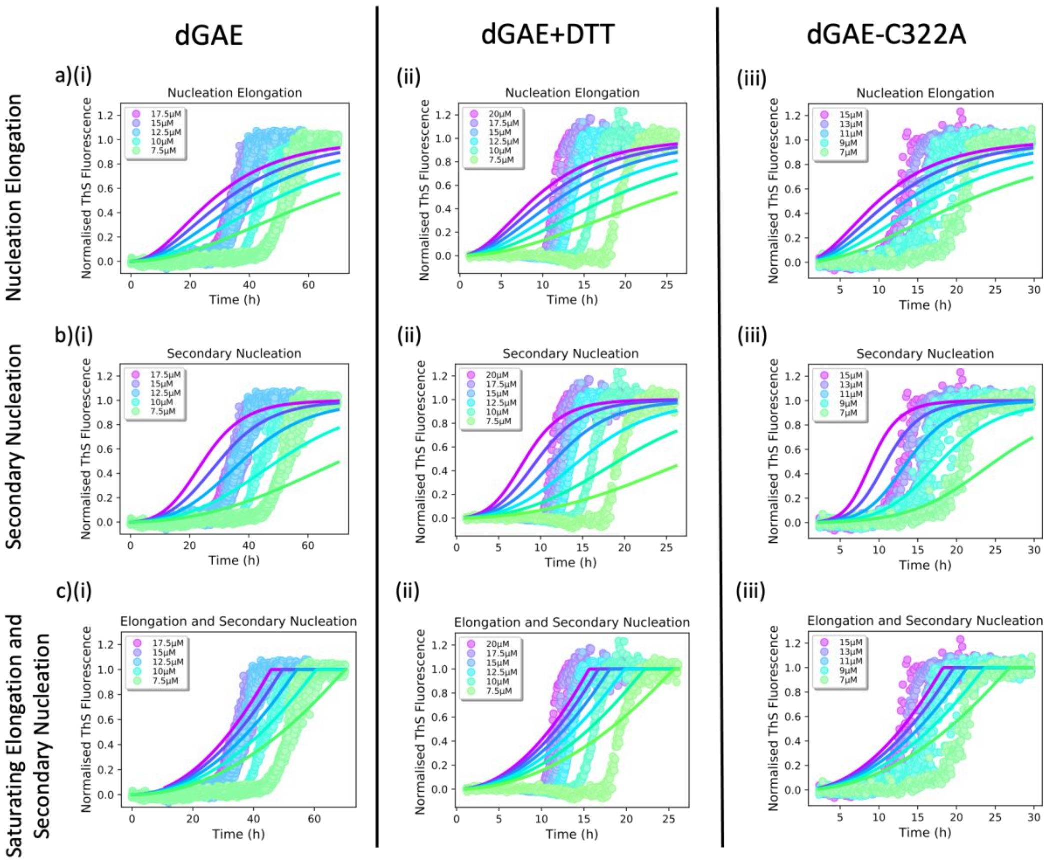

d)

	Assembly condition		
**Aggregation Model**	**dGAE**	**dGAE+DTT**	**dGAE+C322A**
Multi-step Secondary Nucleation	0.00787	0.00587	0.00973
Saturating Elongation and Fragmentation	0.00696	0.00589	0.00996
Nucleation Elongation	0.0628	0.0829	0.0659
Secondary Nucleation	0.0609	0.0804	0.0532
Saturating Elongation and Secondary Nucleation	0.0312	0.0301	0.0201

Supplementary figure 1: Global assembly mechanisms that do not fit dGAE aggregation kinetics. dGAE assembled in non-reduced conditions shown on left section. (i), dGAE assembled in reduced conditions with 10mM DTT in middle section (ii), and dGAE-C322A assembled in non-reduced conditions (iii). Normalised kinetic profiles plotted against different models of assembly:(a) nucleation elongation, (b) secondary nucleation, and (c) saturating elongation and secondary nucleation. Mean residual error (MRE) value for each assembly condition kinetics fitted to the varying aggregation models calculated by Amylofit (d).

Supplementary figure 2.

a)

Dataset	Primary rate at 15µM total protein (s-1)	Secondary rate at 15µM total protein (s-1)
dGAE	3.6E-07	9.2E-05
dGAE-DTT	1.4E-08	4.5E-04
dGAE-C322A	1.2E-06	2.4E-04

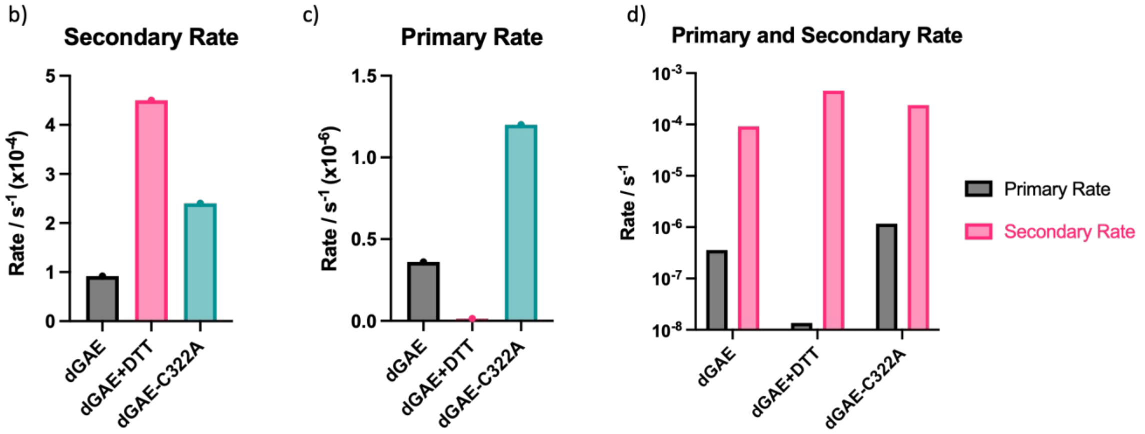

Supplementary Figure 2: The primary and secondary rate constants were obtained from the global fit data shown in figure 1 using Amylofit software using the traces of 15µM experiments. (a). The secondary rate (b) and primary rate (c) are shown separately to illustrate the differences between the assembly conditions and shown all together for comparison between primary and secondary within each assembly conditions.

Supplementary Figure 3.

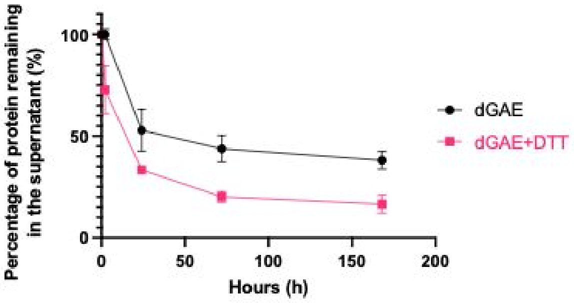

Supplementary Figure 3: Identifying available soluble/unassembled species throughout assembly of dGAE in reducing and non-reducing conditions. To quantify soluble species remaining in the assembly mixture, protein concentration of the supernatant was quantified with a Bichinchoninic acid assay (BCA) and plotted as a percentage of the concentration at 0h. Samples were taken at 0h, 2h, 24h, 72h and 7d. dGAE sample (black): 0h (100.00% ± 0.00%), 2h (100.00% ± 2.60%), 24h (52.87% ± 10.30%), 72h (43.77% ± 6.55%), 7d (168h: 38.10% ± 4.35%) n=3. dGAE+DTT sample (pink): 0h (100.00% ± 0.00%), 2h (72.73% ± 11.75%), 24h (30.24% ± 1.11%), 72h (19.68% ± 3.77%), 7d (168h: 16.53% ± 4.46%) n=4.

Method

dGAE (100µM) diluted in 10mM PB +/- 10mM DTT and incubated at 37°C whilst agitating at a speed of 700rpm on an Eppendorf ThermoMixer® for 7d. The assembly mixture was centrifuged at 16,000g for 30min to separate any aggregated species (pellet) and the soluble protein (supernatant). A small sample of the supernatant was used in a BCA assay (Pierce™ BCA Protein Assay Kits), using the reducing agent-compatible BCA for the dGAE+DTT samples, to quantify the amount of soluble protein left in the assembly mix. This was taken as the amount of unaggregated protein in the sample, which was plotted as a percentage of the concentration at 0h.

Supplementary Figure 4.

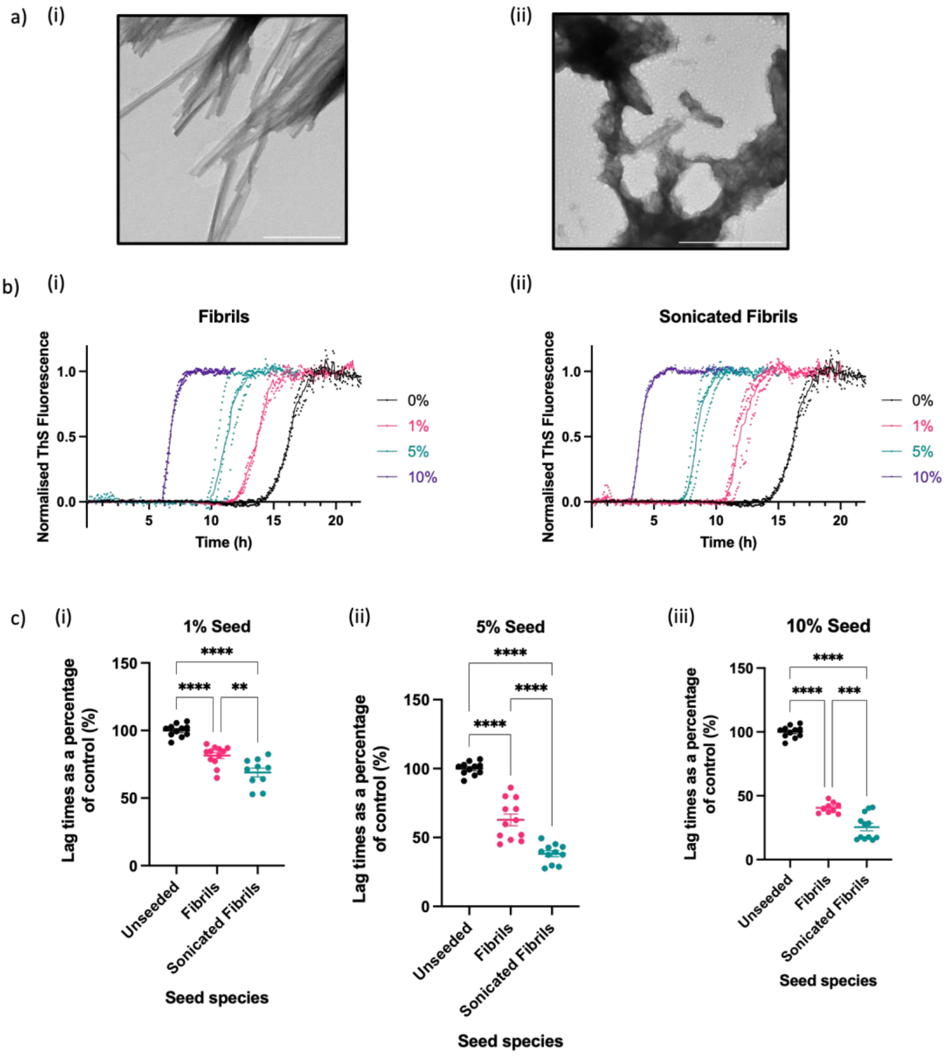

Supplementary Figure 4: Seeding from dGAE-C322A seeds before and after sonication

Electron micrograph of dGAE-C322A fibrils produced with 400µM monomeric dGAE-C322A (ai) and after sonication (aii). Scale bar represents 500nm. Example of normalised thioflavin-S kinetics from a single experiment showing the seeding capability of dGAE-C322A fibrils (bi) and after sonication (bii). 0% (control - black), 1% (pink), 5% (green) and 10% (purple). (ci) Quantification and comparison of the lag times with 1% seeds of each condition as a percentage of the control. One-way ANOVA shows significant difference between groups (F=43.80, R^2=0.7449, $p < 0.0001$) from 3 independent tests. Turkey's multiple comparison test shows sonicated fibril seed (68.92% ± 3.247%) induce a significant reduction in lag phase when compared to the control (100% ± 1.399%, $p < 0.0001$) and fibril seeds (81.46% ± 2.142%, p = 0.0018). Fibril seeds induce a significant reduction in lag phase when compared to control ($p < 0.0001$). (cii) Quantification and comparison of the lag times with 5% seeds of each condition as a percentage of the control. One-way ANOVA shows significant difference between groups (F=110.9, R^2=0.8774, $p < 0.0001$) from 3 independent tests. Turkey's multiple comparison test shows sonicated fibril seed (38.25% ± 2.127%) induce a significant reduction in lag phase when compared to the control (100% ± 1.399%, $p < 0.0001$) and fibril seeds (62.76% ± 4.140%, $p < 0.0001$). Fibril seeds induce a significant reduction in lag phase when compared to control ($p < 0.0001$). (ciii) Quantification and comparison of the lag times with 10% seeds of each condition as a percentage of the control. One-way ANOVA shows significant difference between groups (F=338.6, R^2=0.9589, $p <$ 0.0001) from 3 independent tests. Turkey's multiple comparison test shows sonicated fibril seed

(25.47% ± 2.920%) induce a significant reduction in lag phase when compared to the control (100% ± 1.399%, $p < 0.0001$) and fibril seeds (40.51% ± 1.403%, $p = 0.0002$). Fibril seeds induce a significant reduction in lag phase when compared to control ($p < 0.0001$).

Supplementary Figures 5.

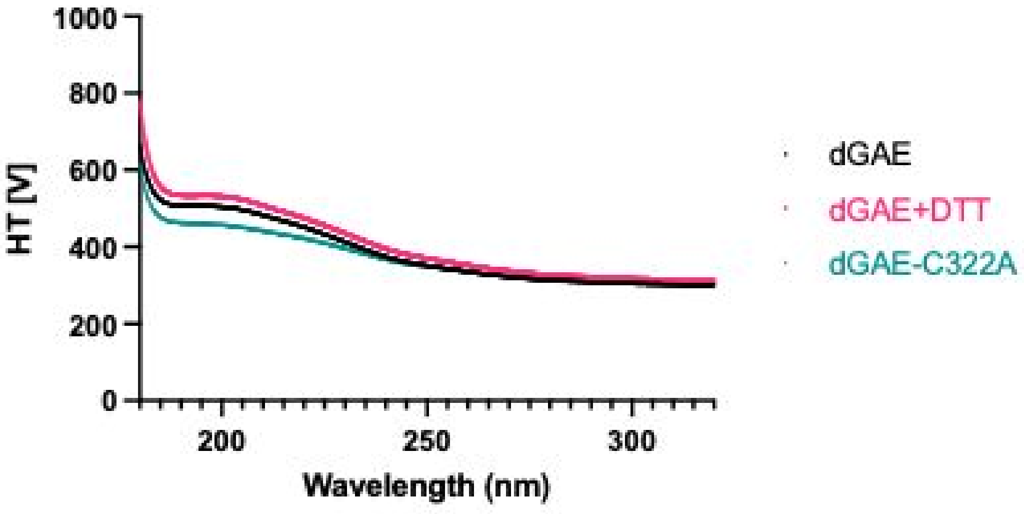

Supplementary Figure 5: HT reading of each fibril sample during CD. HT reading over 180-320nm CD reading of the fibrils performed from each condition, dGAE (black), dGAE+DTT (pink) and dGAE-C322A (green). This shows little difference in HT between the samples and shows that the washes remove any interference that the DTT may introduce.

Supplementary Figure 6.

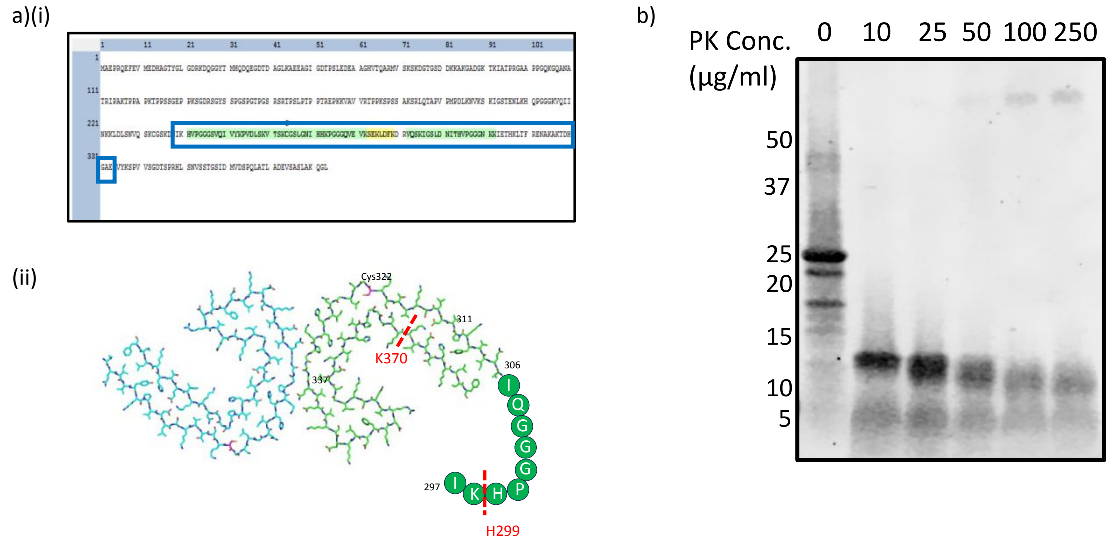

Supplementary figure 6: 12kDa band is resistant to increased protease K concentration.
(ai) Mass spectrometry analysis of the band at 8kDa after protease K treatment. Green region shows the region believed to be protease K resistant in comparison with the whole dGAE sequence indicated with the blue box. (aii) Illustrating the protease K resistant core using the dGAE fold resolved by Lövestam et al., 2022. (b) 200µM dGAE fibrils formed in non-reducing conditions incubated with 0, 10, 25, 50, 100 and 250µg/ml protease K for 1h at 37°C before being analysis with SDS-PAGE and stained with Coomassie protein stain.

Method:
Mass spectrometry carried out to identify the sequence of the 8 kDa band after PK digestion. The band was cut out of the gel and put into water in a microcentrifuge tube. The excised band was sent for tryptic digestion and LC-MS analysis by the Proteomics facility at the University of Bristol [1].

Supplementary Figure 7.

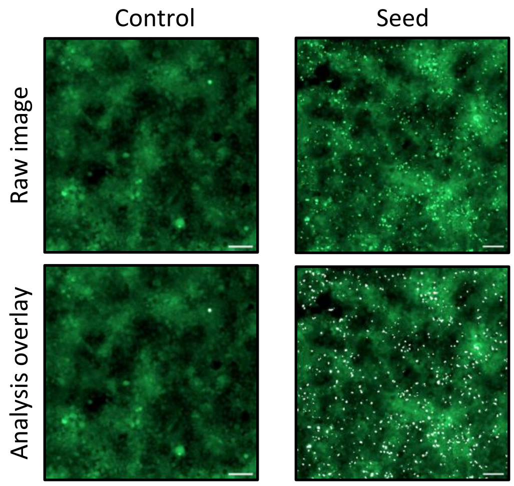

Supplementary Figure 7: Demonstration of image analysis undertaken of FRET Biosensor cells with the Molecular Devices ImageXpress Pico and CellReporterXpress.
Raw images of FRET Biosensor cells treated with phosphate buffer along (control) or with 10µM sonicated dGAE-C322A fibrils (seed), which shows an increase in fluorescence puncta taken with the ImageXpress Pico without the analysis overlay (top row). Images acquired with the analysis overlay undertaken (white signal shows isolated punctate signal) with the described parameters to isolate the punctate signal wanted. All scale bars represent 50µm.

References

1. Bansal A, Schmidt M, Rennegarbe M, Haupt C, Liberta F, Stecher S, et al. AA amyloid fibrils from diseased tissue are structurally different from in vitro formed SAA fibrils. Nature communications. 2021;12(1):1013.
